# Rewiring immune evasion in liver metastases: WNT11 as a central node — a mini review

**DOI:** 10.3389/fonc.2025.1666889

**Published:** 2025-12-04

**Authors:** Xiaoling Wang, Youcai Huang, Tingting Luo, Qinxian Liu, Yu Tian, Xiaoyu Hu, Yi Zheng, Shumin Fang, Yanyang Tu, Haining Zhen, Yu Guo

**Affiliations:** 1 Science Research Center, Huizhou Central People’s Hospital, Huizhou, Guangdong, China; 2 Science Research Center, Huizhou Central People’s Hospital, Guangdong Medical University, Huizhou, Guangdong, China; 3 Huizhou Central People’s Hospital Academy of Medical Sciences, Huizhou, Guangdong, China; 4 Department of Neurosurgery, Xijing Hospital, Fourth Military Medical University, Xian, China; 5 Zhuhai People’s Hospital (The Affiliated Hospital of Beijing Institute of Technology, Zhuhai Clinical Medical College of Jinan University), Zhuhai, China

**Keywords:** liver metastasis, Wnt11, immune checkpoint blockade resistance, CaMKII signaling, tumor immune microenvironment

## Abstract

Liver metastasis (LM) poses a formidable barrier to effective immunotherapy, largely due to its uniquely immunosuppressive microenvironment and resistance to immune checkpoint blockade (ICB).Among emerging mechanisms, WNT11, a non-canonical WNT ligand, has been identified as a preclinical modulator of immune evasion in LM. Acting through a calcium-dependent CAMKII signaling pathway axis, WNT11 suppresses CD8^+^ T-cell recruitment via downregulation of chemokines such as CXCL10 and CCL4 and promotes M2-like macrophage polarization through IL17D induction. This dual mechanism contributes to the formation of an immune-excluded, tolerogenic niche that undermines the efficacy of anti-PD-1 therapies. Targeting the WNT11/CAMKII axis restores immune infiltration and sensitizes LM to ICB in preclinical models, highlighting a promising therapeutic strategy. Although no direct WNT11-targeted therapies are currently available, multiple pharmacological strategies targeting its proximal and downstream effectors—such as FZD/ROR, CAMKII, PKC/JNK/NFAT, and associated crosstalk pathways like TGF-β, IDO1, and myeloid axes—are under active exploration. Additionally, circulating WNT11 levels may also serve as a predictive biomarker for patient stratification and treatment monitoring. Despite challenges related to pathway complexity and tumor heterogeneity, this mini review synthesizes recent advances in understanding the WNT11-driven tumor-immune axis and proposes a translational roadmap for combination strategies to overcome ICB resistance in liver metastasis.

## 1 Introduction

Liver metastasis (LM) remains one of the most challenging clinical manifestations of advanced malignancies, particularly in colorectal, pancreatic, and breast cancers (1, 2). Despite the significant breakthroughs in immune checkpoint blockade (ICB) therapies—especially PD-1/PD-L1 inhibitors—most patients with LM exhibit poor or transient responses, underscoring the profound immune tolerance of the hepatic microenvironment (3, 4). Unlike primary tumors, LM frequently presents an “immune desert” or “immune-excluded” phenotype, characterized by minimal CD8^+^ T-cell infiltration and suppressed cytotoxic responses (5, 6). This immune resistance highlights the need for a deeper mechanistic understanding of how the metastatic liver niche actively impairs anti-tumor immunity.

Among the emerging molecular regulators implicated in this immune dysfunction, WNT11—a non-canonical Wnt ligand traditionally studied in the context of embryogenesis and tissue morphogenesis—has garnered increasing attention (7). Emerging evidence indicates that WNT11 may influence the tumor-immune interface by modulating cytotoxic T=cell infiltration and macrophage polarization (8). Mechanistically, it is speculated to act through the calcium/calmodulin-dependent protein kinase II (CAMKII) pathway (9, 10). Unlike canonical Wnt signaling, which primarily signals through β-catenin stabilization, WNT11 may antagonize β-catenin activity in certain contexts, suggesting intricate crosstalk between non-canonical and canonical Wnt cascades in tumor immunity (11).

Importantly, WNT11 expression and function appear to be highly context-dependent across tumor types. Primary tumors that metastasize to the liver exhibit distinct immunological phenotypes, ranging from “hot” (inflamed) to “cold” (immune-excluded or immune-desert) TMEs. In colorectal cancer, microsatellite-stable (MSS) and microsatellite instability-low (MSI-L) subtypes are typically characterized by poor T-cell infiltration and dense stromal barriers, consistent with a “cold” TME (12). These tumors are resistant to immune checkpoint blockade and are enriched in WNT/β-catenin pathway activity, which has been implicated in excluding Batf3^+^ dendritic cells and impairing CD8^+^ T-cell priming. Canonical WNT signaling in these settings promotes immune exclusion by downregulating chemokines such as CCL4 and CXCL10, and reducing DC recruitment to the tumor bed. In contrast, MSI-H colorectal cancers generally exhibit “hot” immune profiles, with high CD8^+^ T-cell infiltration and increased responsiveness to PD-1 blockade. Notably, WNT11 expression has been observed in some “cold” MSS CRCs (13), where it may contribute to non-canonical immune suppression by dampening NF-κB–driven cytokine responses and promoting M2 macrophage polarization. These tumor-intrinsic WNT signals—canonical or non-canonical—may thus define key immune evasive strategies even in primary sites, prior to liver colonization.

In colorectal cancer (CRC), pancreatic ductal adenocarcinoma (PDAC), and breast cancer—the major sources of LM—WNT11 has been associated with epithelial–mesenchymal transition (EMT), matrix remodeling, and stromal reprogramming (14). These processes converge on both immune evasion and metastatic competence. In addition to its roles in metastatic and tumour contexts, WNT11 has been implicated in several inflammatory and tissue-homeostatic processes. For example, WNT11 was shown to suppress intestinal epithelial cell invasion and dampen bacterial-induced inflammation in the gut epithelium, indicating a link to innate immunomodulation (14). Across different tumor types, non-canonical Wnt11 signalling correlates with enhanced epithelial-mesenchymal transition (EMT) and invasive phenotypes in colorectal, pancreatic and other cancers, suggesting its tumor-type-specific immuno-remodelling potential. Moreover, in the normal liver, WNT11 is enriched in the perivenular (zone-3) hepatocytes and contributes to hepatic zonation and regenerative processes, pointing to a physiological role in liver homeostasis that may predispose the hepatic environment to immune tolerance (15, 16). Furthermore, beyond cancer, WNT11 plays a pivotal role in maintaining hepatic homeostasis and regenerative capacity. It influences the activation of liver sinusoidal endothelial cells (LSECs), Kupffer cells, and hepatic progenitor niches during injury repair, all of which are essential components of the immunoregulatory landscape in both normal and pathological liver settings. These non-malignant functions of WNT11 may partly explain its immunosuppressive potency in liver metastases.

The hepatic immune microenvironment imposes a unique layer of complexity on WNT11-mediated signaling. Liver sinusoidal endothelial cells (LSECs) contribute to an intrinsic tolerogenic milieu by expressing PD-L1, secreting IL-10, and presenting antigens in a non-inflammatory fashion (17, 18). Kupffer cells, the resident macrophages of the liver, are pre-conditioned toward an anti-inflammatory state and rapidly secrete TGF-β and prostaglandins upon activation, further dampening cytotoxic T-cell responses (19). Hepatic stellate cells (HSCs), upon stimulation by WNT and TGF-β cues, release retinoic acid and CXCL12, which recruit regulatory T cells and myeloid-derived suppressor cells (MDSCs) (20). Together, these cellular networks provide a fertile ground for WNT11 to reinforce immune tolerance—explaining why identical WNT cues that promote inflammation in peripheral tissues may instead induce immune suppression within the liver.

In this context, Jiang et al. (2025) have recently provided a foundational study highlighting the role of WNT11 in promoting CD8^+^ T-cell exclusion and resistance to anti-PD-1 therapy in liver metastases (7). While that work focused primarily on intratumoral immune evasion within hepatic lesions, our review aims to extend this understanding by integrating WNT11’s broader mechanistic roles—including its signaling crosstalk with β-catenin, tissue-specific functions in CRC, PDAC, and breast cancers, and immunological influence in liver regeneration niches such as LSECs and Kupffer cells (21).

This review therefore focuses on the emerging WNT11/CAMKII axis as a newly identified immune resistance pathway in liver metastasis, and discusses its mechanistic roles, clinical implications, and therapeutic potential in reshaping the metastatic tumor microenvironment.

## 2 The WNT11/CAMKII signaling axis: a driver of immune evasion in LM

**Figure 1:**
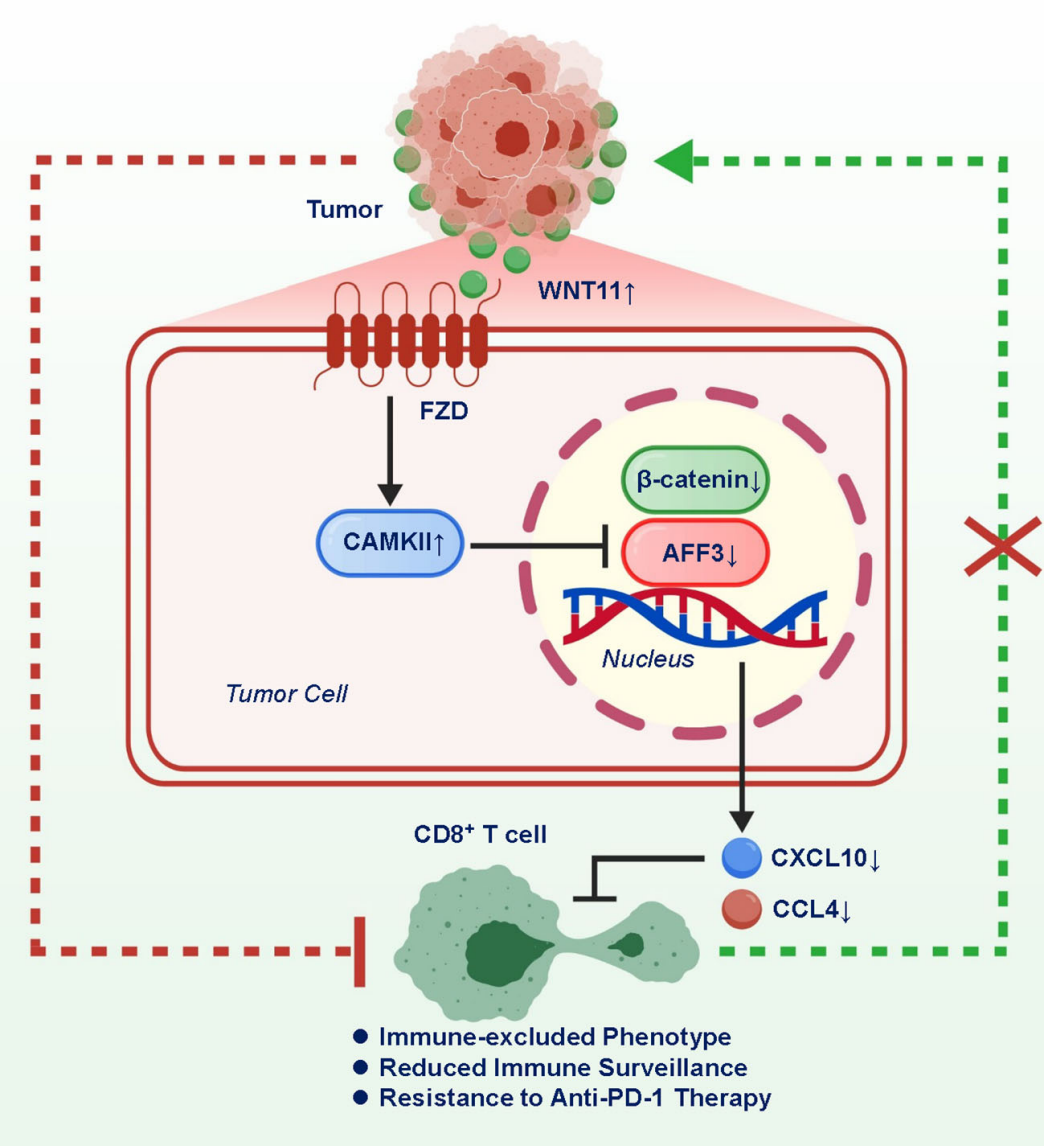
Mechanistic illustration of the WNT11/CAMKII signaling axis driving CD8^+^ T-cell exclusion in liver metastasis.

WNT11’s role in modulating the tumor microenvironment (TME) has gained considerable attention due to its dual capacity to influence both stromal and immune components (7, 22, 23). Mechanistically, WNT11 exerts its immunosuppressive effects through the activation of the calcium/calmodulin-dependent protein kinase II (CAMKII) pathway. This activation leads to the downregulation of β-catenin and its transcriptional coactivator AFF3, resulting in the suppression of chemokines such as CXCL10 and CCL4—key mediators for CD8^+^ T-cell recruitment. As a consequence, tumors overexpressing WNT11 display a significant reduction in cytotoxic T-cell infiltration, creating an “immune-excluded” TME. This phenotype prevents effective immune surveillance and facilitates tumor progression under immunotherapeutic pressure. The inhibition of CXCL10 and CCL4 not only limits T-cell chemotaxis but also reshapes the cytokine milieu toward a more tolerogenic landscape. Notably, the repression of β-catenin activity by WNT11 contrasts with canonical WNT signaling, highlighting the context-specific nature of WNT pathway interactions in cancer. These findings position the WNT11/CAMKII/β-catenin-AFF3 axis as a novel immune checkpoint regulator in LM (**Figure 1**).

## 3 IL17D-mediated macrophage polarization and immune suppression

**Figure 2:**
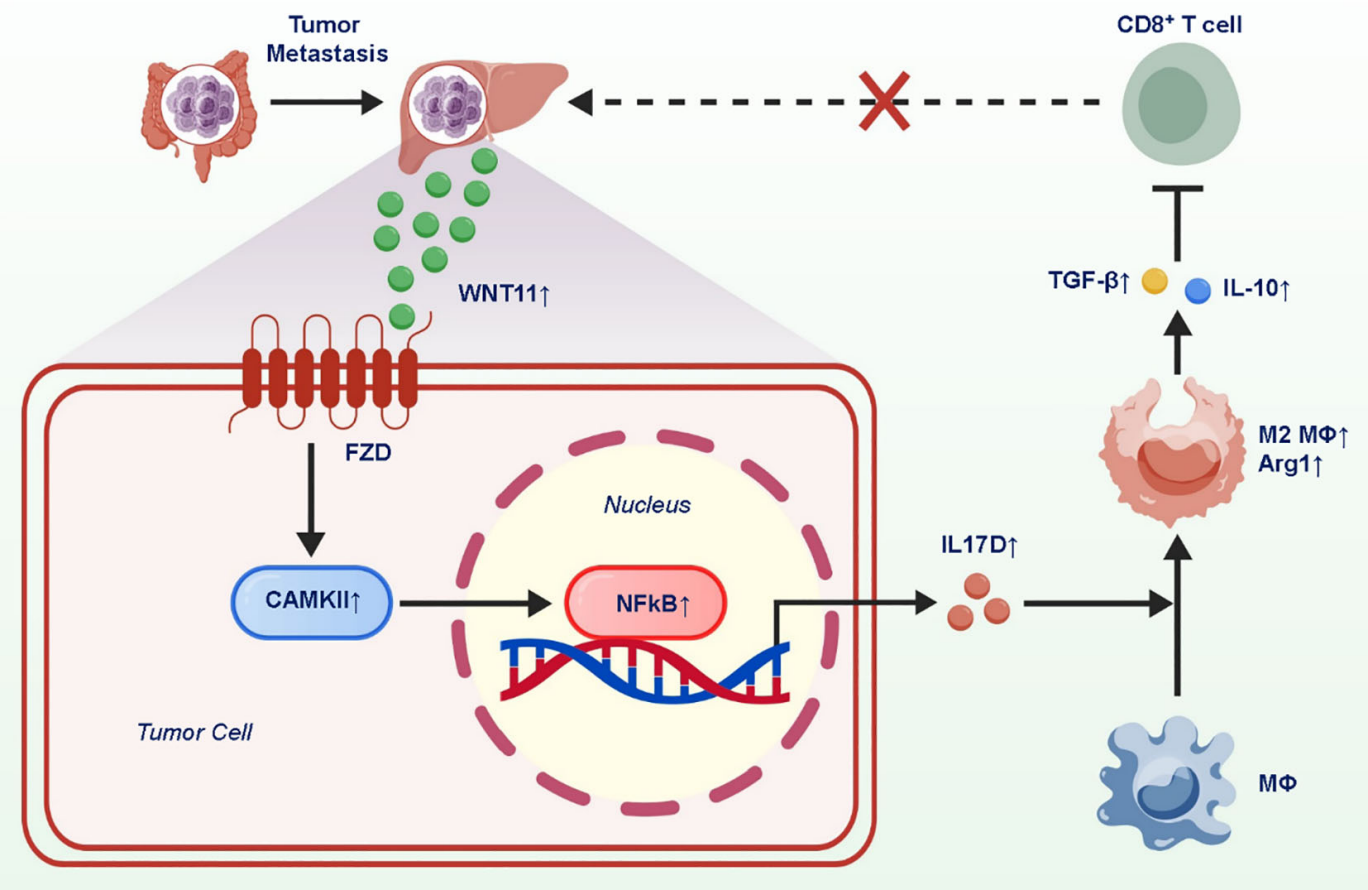
WNT11-induced IL17D expression promotes M2-like macrophage polarization and immune suppression in liver metastasis.

In addition to blocking T-cell entry, WNT11 reinforces local immune suppression through macrophage modulation. Specifically, WNT11 activates CAMKII-dependent NF-κB signaling, leading to the upregulation of interleukin-17D (IL17D). This cytokine plays a pivotal role in promoting the polarization of tumor-associated macrophages (TAMs) toward an M2-like immunosuppressive phenotype. These macrophages secrete anti-inflammatory cytokines, inhibit T-cell activation, and facilitate tumor immune escape. This dual immune evasion mechanism—T-cell exclusion and macrophage-induced suppression—creates a formidable barrier against immune checkpoint therapies. Unlike traditional M2 inducers such as IL-4 or CSF1, the WNT11–IL17D axis suggests a tumor-intrinsic program capable of orchestrating macrophage function independently of stromal inputs (7). The identification of this axis also raises intriguing questions regarding the crosstalk between WNT signaling and innate immune reprogramming. Together, these findings delineate a two-tiered suppression model, wherein WNT11 acts as a master regulator of both adaptive and innate immune components in the liver metastatic niche (**Figure 2**).

## 4 Therapeutic implications: targeting the WNT11/CAMKII axis

**Figure 3:**
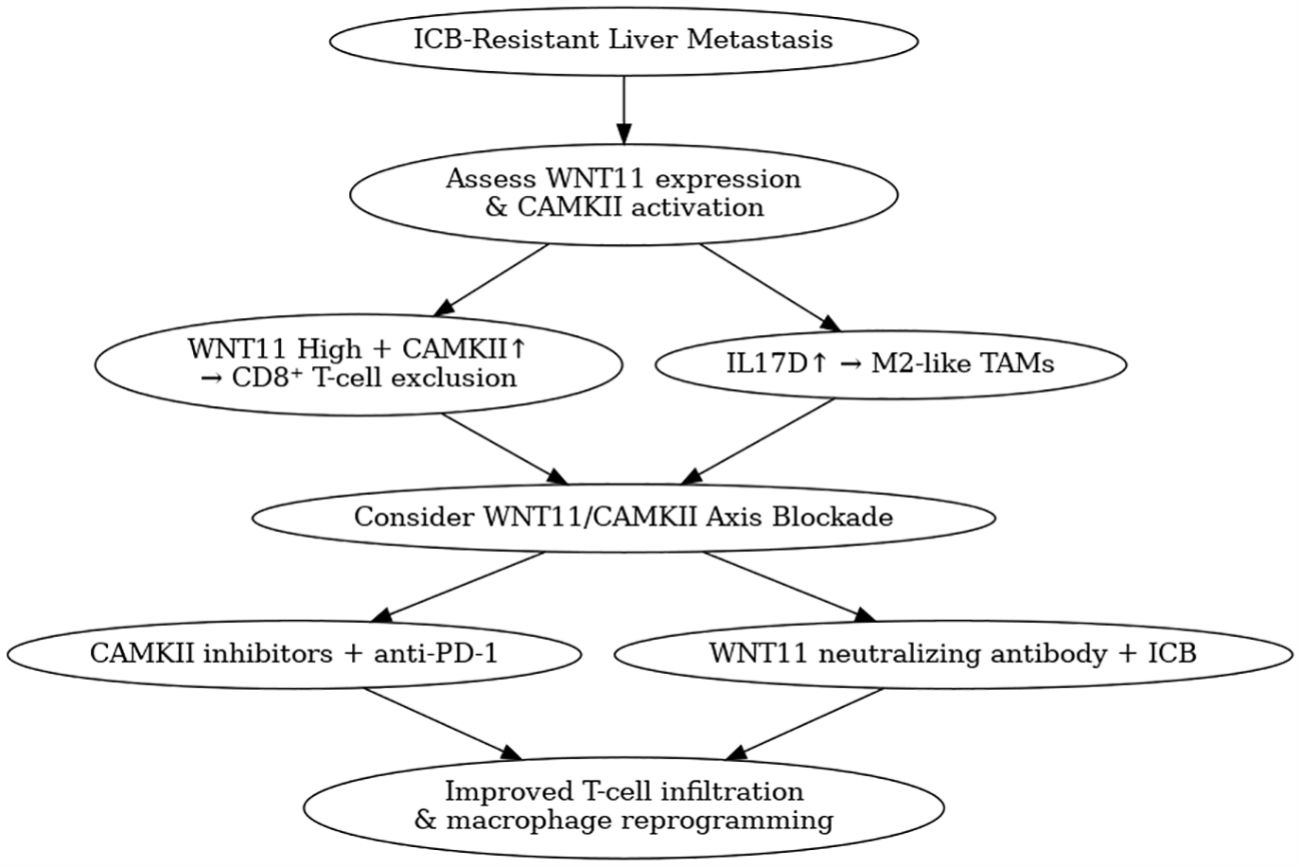
Therapeutic roadmap for targeting the WNT11/CAMKII axis in liver metastasis.

The aemerging role of WNT11 as a modulator of immune exclusion and suppression presents compelling opportunities for therapeutic intervention. In murine models of LM, pharmacological inhibition of CAMKII significantly restores CD8^+^ T-cell infiltration and enhances the efficacy of anti-PD-1 therapy. This synergy suggests that WNT11/CAMKII blockade may sensitize otherwise non-responsive LM tumors to immunotherapy. Furthermore, the accessibility of CAMKII inhibitors, some of which are already under investigation for neurodegenerative diseases and cardiac dysfunctions, could accelerate the translational application of these findings. Given that WNT11 is enriched in perivenular (zone 3) hepatocytes and contributes to hepatic regeneration and immune tolerance , combination therapies targeting WNT11/CaMKII alongside ICB may exploit the liver’s regenerative-immune balance to enhance therapeutic response (24). Another promising aspect is the identification of WNT11 as a circulating biomarker. Elevated serum WNT11 levels correlate with poor prognosis and immune exclusion signatures in patients with colorectal cancer LM, indicating its potential utility as a minimally invasive diagnostic and predictive tool. This could enable better patient stratification and real-time monitoring of immunotherapy response. To facilitate clinical decision-making, we propose a conceptual roadmap integrating diagnostic markers (e.g., serum WNT11 levels, CAMKII phosphorylation) with potential combination strategies (**Figure 3**), including axis inhibitors alongside ICB.

**Figure 4:**
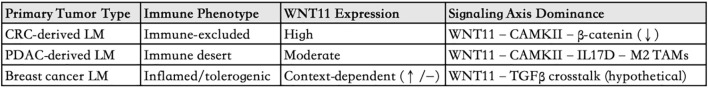
Tumor–immune–signaling matrix in liver metastases This table summarizes the proposed heterogeneity of WNT11-mediated immunosuppression in liver metastases originating from colorectal cancer (CRC), pancreatic ductal adenocarcinoma (PDAC), and breast cancer. It integrates immune phenotypes (e.g., immune-excluded or desert), WNT11 expression levels, and hypothesized dominant downstream signaling axes, including CAMKII-β-catenin repression and IL17D-mediated M2 macrophage polarization.

Future clinical trials evaluating combination strategies—targeting both immune checkpoints and the WNT11/CAMKII axis—are warranted to fully exploit this new avenue in personalized cancer immunotherapy. To better conceptualize the heterogeneity in WNT11-driven immune resistance across different liver metastasis origins, we propose a tumor–immune–signaling matrix (**Figure 4**), integrating primary tumor source, immune phenotypes, and dominant WNT11 downstream effectors. As shown in **Table 1**, the dominant WNT ligands, co-receptors, downstream effectors, and corresponding immune phenotypes vary substantially across different liver-metastatic lineages, highlighting the heterogeneity of WNT-driven immune evasion.

**Table 1:** Dominant WNT axes and immune phenotypes across liver-metastatic lineages.

Primary tumor origin	Dominant WNT ligand(s)/axis	Co-receptors	Key downstream effectors	Reported immune phenotype in LM	Representative references
Colorectal cancer (CRC)	WNT11 → CAMKII → β-catenin↓ / CXCL10↓	FZD7 / ROR2	AFF3, NF-κB	CD8^+^ T-cell exclusion; M2 TAM enrichment	Jiang et al., 2025 (7); Menck et al., 2021 (38)
Pancreatic ductal adenocarcinoma (PDAC)	WNT5A / WNT11 → Ca²^+^–NFAT	ROR1 / RYK	IL17D	TAM polarization; desmoplastic barrier	Zhang et al., 2022 (39)
Breast cancer	WNT11 → JNK / PCP pathway	FZD6 / ROR2	c-Jun, DAAM1	Stromal remodeling; immune-cold TME	Menck et al., 2021 (38); Johannes et al., 2023 (40)
Melanoma (LM)	WNT5A → β-catenin / PKC hybrid	FZD5	MITF, AXL	T-cell exclusion; PD-L1 ↑	Kathryn et al., 2019 (41)

**Figure 5:**
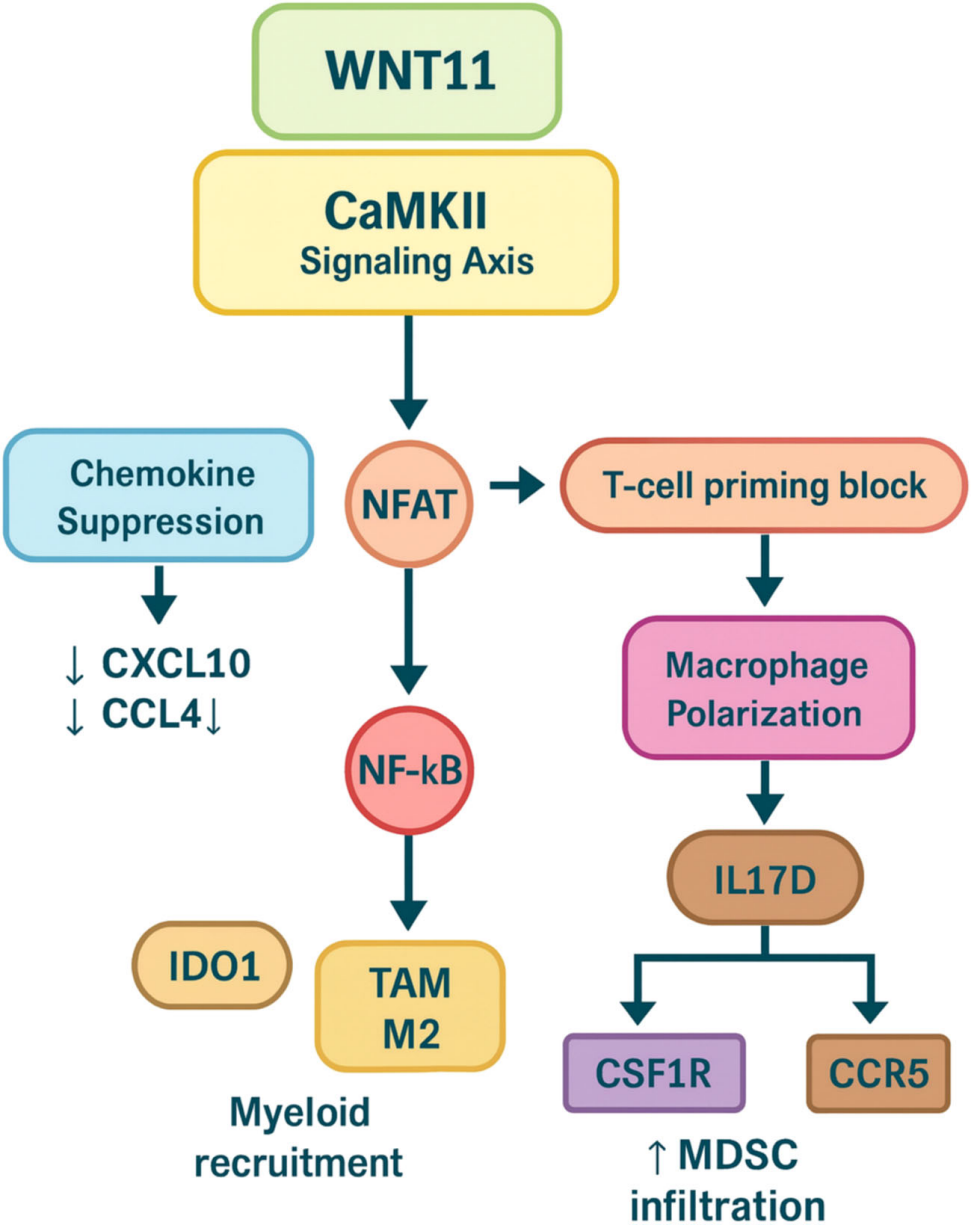
Convergent immunosuppressive activities of WNT11/CaMKII axis.

To better conceptualize the therapeutic potential of WNT11 modulation, we propose a signaling schema that highlights its proximal axis (FZD/ROR→DVL→PLCβ→Ca²^+^→CAMKII/PKC/JNK/NFAT) and potential immunomodulatory intersections. These include its known antagonism of β-catenin, cooperative crosstalk with TGF-β signaling, suppression of chemokine gradients (e.g., CXCL10, CCL4), and contributions to macrophage polarization via IL17D induction. Additionally, WNT11 may interact with key metabolic immune barriers, such as IDO1 activity, and interface with myeloid recruitment axes including CSF1R–CCR2/CCR5, further reinforcing immune tolerance within the hepatic niche. These convergent or parallel signals underscore WNT11’s potential as a candidate modulator—rather than a singular driver—within a broader immunosuppressive landscape. A visual comparison of these pathways is provided in **Figure 5**.

## 5 Therapeutic opportunities and druggable nodes within the WNT11 axis

Although no therapies currently exist that directly inhibit WNT11, its immunomodulatory network presents multiple pharmacologically tractable entry points. These include proximal receptors (e.g., FZD and ROR), downstream Ca²^+^-dependent mediators (e.g., DVL, CAMKII, PKC, JNK, NFAT), and crosstalk effectors involved in metabolic and myeloid suppression loops (e.g., TGF-β, IDO1, CSF1R-CCR2/CCR5).

Notably, several agents targeting these nodes have entered preclinical or clinical development in cancer and other diseases. For example, the anti-Frizzled monoclonal antibody Vantictumab (OMP-18R5) has completed Phase Ib trials in solid tumors (NCT01973309). ROR2-targeted antibodies (e.g., LY-3107625) and small-molecule inhibitors (e.g., KY-0105) are undergoing preclinical evaluation.

In the Ca²^+^-dependent branch, CaMKII inhibitors such as SHP-915 and KN-93 have demonstrated efficacy in cardiac and neurological models and could be repurposed for immune-oncology applications. JNK inhibitor CC-930 (Tanzisertib) has advanced to Phase II trials in idiopathic pulmonary fibrosis (NCT01203943), while PKC inhibitor Enzastaurin (LY317615) has reached Phase III in lymphoma (NCT00319535). NFAT signaling can be modulated via clinically approved calcineurin inhibitors such as Cyclosporine A, widely used in transplant immunosuppression.

Beyond the canonical WNT11-Ca²^+^ axis, several agents targeting metabolic and myeloid barriers may hold indirect therapeutic relevance. The IDO1 inhibitor Epacadostat (INCB024360) reached Phase III in melanoma (NCT02752074), highlighting the feasibility of metabolic reprogramming to reverse T-cell exhaustion. Meanwhile, myeloid-directed therapies such as the CSF1R inhibitor Pexidartinib (FDA-approved for tenosynovial giant cell tumor), CCR5 antagonist Maraviroc (approved for HIV), and the dual CCR2/CCR5 antagonist BMS-813160 (Phase II, metastatic CRC; NCT03184870) offer promising options to curb macrophage-driven immune tolerance. These examples, summarized in **Table 2**, constitute a translational toolbox for indirect modulation of WNT11 signaling—either by impeding upstream ligand–receptor interactions, suppressing the CaMKII-PKC/JNK-NFAT axis, or disrupting cooperating immunosuppressive circuits.

**Table 2:** Representative pharmacological agents targeting the WNT11-associated signaling network and their current development status.

Target node	Representative agents	Development status
FZD	Vantictumab (OMP-18R5)	Phase I (solid tumors)
ROR2	Monoclonal antibodies (preclinical)	Preclinical
DVL	NSC668036 (DVL–PDZ disruptor)	Preclinical
CAMKII	KN-93, GS-680	Preclinical
PKC	Enzastaurin	Phase II (various cancers)
JNK	CC-401, AS602801	Phase I/II
NFAT	VIVIT peptide, FK506	Preclinical/approved (for other uses)
TGF-β	Galunisertib (LY2157299)	Phase II/III
IDO1	Epacadostat	Phase III
CSF1R	Pexidartinib	FDA approved
CCR2/CCR5	BMS-813160/Maraviroc	Phase II/approved (HIV)

While several agents are under development or approved in other disease contexts, liver-specific safety and immunological off-target effects remain a key consideration, particularly in hepatically enriched WNT settings. Future clinical designs should therefore incorporate biomarker-guided patient stratification (e.g., serum WNT11 levels, immune exclusion signatures) and explore combination regimens with ICB to optimize immunological remodeling in liver metastasis.

## 6 Challenges and future directions

While the identification of the WNT11/CAMKII axis represents a promising step toward overcoming immunotherapy resistance in LM, several challenges remain before these findings can be fully translated into clinical benefit. First, the context-dependent nature of WNT signaling poses a major obstacle. WNT ligands often exhibit pleiotropic and sometimes contradictory roles depending on tissue type, tumor origin, and disease stage (25, 26). It is unclear whether targeting WNT11 might inadvertently disrupt physiological processes, particularly in organs with active WNT signaling such as the liver and gut. Therefore, selective targeting strategies—such as tumor-specific delivery systems or context-specific inhibitors—are urgently needed to minimize off-target effects. Second, the heterogeneity of the tumor microenvironment (TME) across patients and metastatic sites must be carefully considered. While WNT11 is implicated in CD8^+^ T-cell exclusion and macrophage polarization in LM, its expression levels and downstream signaling strength may vary significantly across tumor types and individuals (27, 28). Stratifying patients based on WNT11 expression, CAMKII activation, or TME immunotype may be necessary to identify those most likely to benefit from targeted therapy. Third, although CAMKII inhibitors have shown efficacy in preclinical models, their safety, pharmacokinetics, and immunological effects in human cancer patients remain largely unexplored (29–31). Rigorous preclinical testing in combination with immune checkpoint blockade, followed by well-designed early-phase clinical trials, will be critical for assessing the translational feasibility of WNT11-targeted approaches. Finally, the role of WNT11 beyond the liver warrants investigation. Whether WNT11 drives similar immune evasion in other metastatic niches such as the lung, peritoneum, or brain is unknown. Expanding research into WNT11-mediated immune remodeling in diverse tumor contexts could uncover shared or unique resistance mechanisms, broadening the therapeutic scope of this target. In sum, while promising, targeting the WNT11/CAMKII axis requires careful navigation of biological complexity and clinical variability. Addressing these challenges through integrative translational research will be essential to unlock its full potential in cancer immunotherapy.

## 7 Conclusion

The identification of the WNT11/CAMKII signaling axis as an emerging modulator of immune evasion and immunotherapy resistance in LM represents a promising preclinical insight in cancer immunology. This pathway not only orchestrates the exclusion of cytotoxic CD8^+^ T cells from the tumor microenvironment but also drives the polarization of TAMs toward an immunosuppressive phenotype (32, 33). Such dual immunomodulatory capacity positions WNT11 as a candidate convergent node rather than a singular driver in reshaping the immune landscape of metastatic lesions, particularly within the liver, which is inherently an immunotolerant organ. While current evidence is predominantly derived from preclinical models, these findings warrant further clinical validation to clarify WNT11’s translational relevance. This insight opens up new avenues for therapeutic intervention. Targeting the WNT11/CAMKII axis could simultaneously reverse immune exclusion and reprogram macrophages to support anti-tumor immunity, thereby overcoming two major bottlenecks in immunotherapy. Furthermore, the liver’s unique vascular and stromal context, which often limits immune cell infiltration and promotes immune suppression, may be particularly susceptible to interventions that recalibrate the WNT11-mediated immune network. As the field of immuno-oncology matures, it is increasingly evident that monotherapies—especially those focused solely on ICB—are insufficient in many metastatic settings (34, 35). The integration of WNT11-targeted strategies could synergize with existing modalities such as PD-1/PD-L1 inhibitors, CAR-T cell therapy, or tumor vaccines, to produce more durable and systemic immune responses (36, 37). Additionally, WNT11 and its downstream effectors could serve as predictive biomarkers, enabling personalized treatment approaches and early identification of resistance mechanisms. Future studies should aim to dissect the temporal and spatial dynamics of WNT11 expression across metastatic niches, as well as its interaction with other signaling pathways such as TGF-β, β-catenin-independent WNT cascades, and metabolic checkpoints. Preclinical and clinical validation of WNT11 inhibitors, including small molecules, neutralizing antibodies, or RNA-based therapeutics, will be critical in translating these findings into tangible patient benefits. In sum, targeting the WNT11/CAMKII axis offers a compelling strategy to rewire the tumor-immune interface in liver metastasis. Such approaches not only hold promise for expanding the efficacy of current immunotherapies but may also redefine how we understand and exploit immunological vulnerabilities in metastatic cancer.
